# Association of *SOX6* gene polymorphisms with Kashin-Beck disease risk in the Chinese Han population

**DOI:** 10.1515/med-2023-0883

**Published:** 2024-01-06

**Authors:** Na He, Aiwen Hong, Kun Zhao, Zhefan Zhang, Shengli Wang, Yaofei Jia

**Affiliations:** People’s Hospital of Changwu County, Zhaoren Street, Xianyang, Shaanxi 713600, China

**Keywords:** Kashin-Beck disease, risk, *SOX6*, SNPs, case–control study

## Abstract

Kashin-Beck disease (KBD) is an endemic osteochondropathy. A specific gene called SRY-box transcription factor 6 (*SOX6*) is important for forming cartilage. This study aims to explore the potential correlation between *SOX6* single nucleotide polymorphisms (SNPs) and KBD risk for the first time. In the case–control study, 735 unrelated Chinese Han individuals were enrolled. The four mutation sites of the *SOX6* gene (rs4539287 G/A, rs3203295 C/A, rs7928675 C/A, and rs10832681 A/G) were screened and genotyped on the Agena MassARRAY platform. The correlation between *SOX6* SNPs and KBD risk was explored based on logistic regression analysis. The interaction between SNP and SNP was analyzed based on the multi-factor dimensionality reduction (MDR) method. Overall analysis revealed a remarkable correlation between rs7928675 and rs10832681 and the reduction of KBD risk (*p* < 0.05). Subgroup analyses further indicated that these two SNPs have a significant protective effect on KBD risk among participants aged ≤65 years, males, and non-smokers (*p* < 0.05). MDR displayed a marked interaction between rs3203295 and rs10832681. Our study revealed that *SOX6* rs7928675 and rs10832681 are markedly correlated with a reduced risk of KBD in the Chinese Han population, providing a new direction for the prevention, diagnosis, and treatment of KBD.

## 1 Introduction

Kashin-Beck disease (KBD) is an endemic, chronic, degenerative osteoarthropathy that is common in children and adolescents, characterized by joint pain, swelling, and stiffness, and it can lead to chondrocyte necrosis, joint deformities, and joint functional damage in severe cases [1,2]. KBD is quite common worldwide and mainly distributed in the low-selenium (Se) zone of China, which runs from northeast to southwest [3,4]. According to *China Health and Family Planning Statistical Yearbook 2016*, KBD may pose a threat to over 1.16 million individuals residing in 337 counties across 13 provinces of China [5]. Previous studies have suggested that KBD is caused by a combination of genetic and environmental factors [6]. Related studies further revealed a certain correlation between some susceptibility gene variants and the KBD risk, which may be closely related to the occurrence and development of KBD [6–8]. Zhang et al. found a significant correlation between the *ABI3BP* variants (CNV452, rs9850273, and rs7613610) and KBD risk based on a genome-wide copy number variation study and association analysis involving 2,743 Chinese Han adults [9]. Wu et al. conducted an association analysis of genetic association, messenger ribonucleic acid (mRNA), and protein expression of the *ATG4C* gene in KBD patients. The study revealed that *ATG4C* was a novel autophagy-related susceptibility gene of KBD, and its genetic variants (rs11208030, rs4409690, rs12097658, and rs6587988) were significantly associated with KBD risk [10]. Yu et al. evaluated the correlation between selenoprotein gene polymorphism and KBD risk as of January 2021 through meta-analysis. The study found that only *DIO2* (rs225014), *SEPS1* (−105G > A), and *Sep15* (rs5859) gene polymorphisms were significantly associated with KBD risk, while *GPX1* (rs1050450, rs1800668, rs3811699), *DIO2* (rs225014, rs1352815, rs1388382), *TrxR2* (rs1139793, rs5746841), glutathione peroxidase 4 (*GPX4*) (rs713041, rs4807542), and selenoprotein P (*SEPP1*) (rs757925191g/a) did not show statistical significance with KBD risk [11]. As of now, the pathogenesis of KBD remains elusive and the genetic risk of KBD explained by reported susceptibility genes is relatively limited. Therefore, continuing to develop more genetic variation sites related to KBD will be crucial for the prevention, diagnosis, and precise treatment of KBD.

SRY-box transcription factor 6 (*SOX6*), located on chromosome 11, encodes a protein that is a transcriptional activator required for proper development of the central nervous system, cartilage formation, and maintenance of heart and skeletal muscle cells [12,13]. It was reported that *SOX6* is necessary for effective cartilage formation, as its inactivation can affect the differentiation of chondrocytes and neuronal cells, resulting in mild bone defects and bone-related disorders like Tolchin-Le Caignec syndrome, osteoporosis, and osteochondroma [14,15]. Recent studies have revealed that *SOX6* variants can cause neurological syndromes related to hyperactivity disorder, cranial osteoporosis, and osteochondroma [16]. Meanwhile, *SOX6* is a multi-effector gene in osteoporosis, and there is a latent interaction between its multiple genetic mutations and the risk of osteoporosis [17,18]. However, the occurrence of KBD disease is closely related to chondrocyte destruction, but there are currently no reports on the potential relationship between *SOX6* polymorphism and KBD risk.

In this research, we conducted a case–control study on 352 KBD patients and 383 normal individuals in the Chinese Han population, and for the first time evaluated the correlation between *SOX6* single nucleotide polymorphisms (SNPs) (rs4539287, rs3203295, rs7928675, and rs10832681) and the risk of KBD, providing a novel biomarker for the prevention, diagnosis, and treatment of KBD in the future.

## 2 Materials and methods

### 2.1 Study participants

This case–control study enrolled 352 KBD patients and 383 normal individuals from the People’s Hospital of Changwu County. Among them, all patients were diagnosed with KBD through clinical and radiological examination of the skeletal system, and patients with primary osteoarthritis, rheumatoid arthritis, and a family history of joint diseases were excluded. The control group was randomly selected and underwent radiation testing, with no KBD, no osteoarthritis, and no family history of cancer. Prior to the experiment, all subjects were aware of this research purpose and process.


**Ethics approval and consent to participate:** The study was approved by the ethics committees of the People’s Hospital of Changwu County (ethics committee registration number: 2022-A03).

### 2.2 DNA extraction and genotyping of SNPs

Screen and genotype four SNPs of the *SOX6* gene as candidate mutation sites, including rs4539287, rs3203295, rs7928675, and rs10832681. The screening process involves three key steps: (1) obtaining the polymorphism data of the *SOX6* gene from the 1,000 genome project database, (2) using Haploview v 4.2 to screen SNPs, the filtering threshold is Hardy–Weinberg equilibrium (HWE) >0.01 and minor allele frequency (MAF) >0.05, and (3) combining primer design and random selection to further screen SNPs.

Under fasting conditions, we collected fresh venous blood (5 mL) from all subjects and used the DNA extraction kits (GoldMag Biotechnology) to extract genomic DNA. Then, the Nanodrop 2000 spectrophotometer (Thermo, USA) was used to determine DNA concentration and purity. Afterward, four candidate SNPs were genotyped on the Agena MassARRAY platform (Agena Bioscience, USA) and the data were processed using Agena Typer v 4.0 software, with the primer sequence information displayed in Table S1.

### 2.3 Statistical analysis

Using G*Power v 3.1.9.7 to calculate sample size through independent samples *t*-test. Based on SPSS v 25.0 (SPSS Inc., Chicago, IL, USA), the basic characteristics of the case group and control group were analyzed using independent samples *t*-test or chi-squared test. In the control group, the chi-square test analyzed the genotype frequency distribution of candidate SNPs, with *p* > 0.05 indicating that the genotype frequency conformed to HWE.

**Figure 1:**
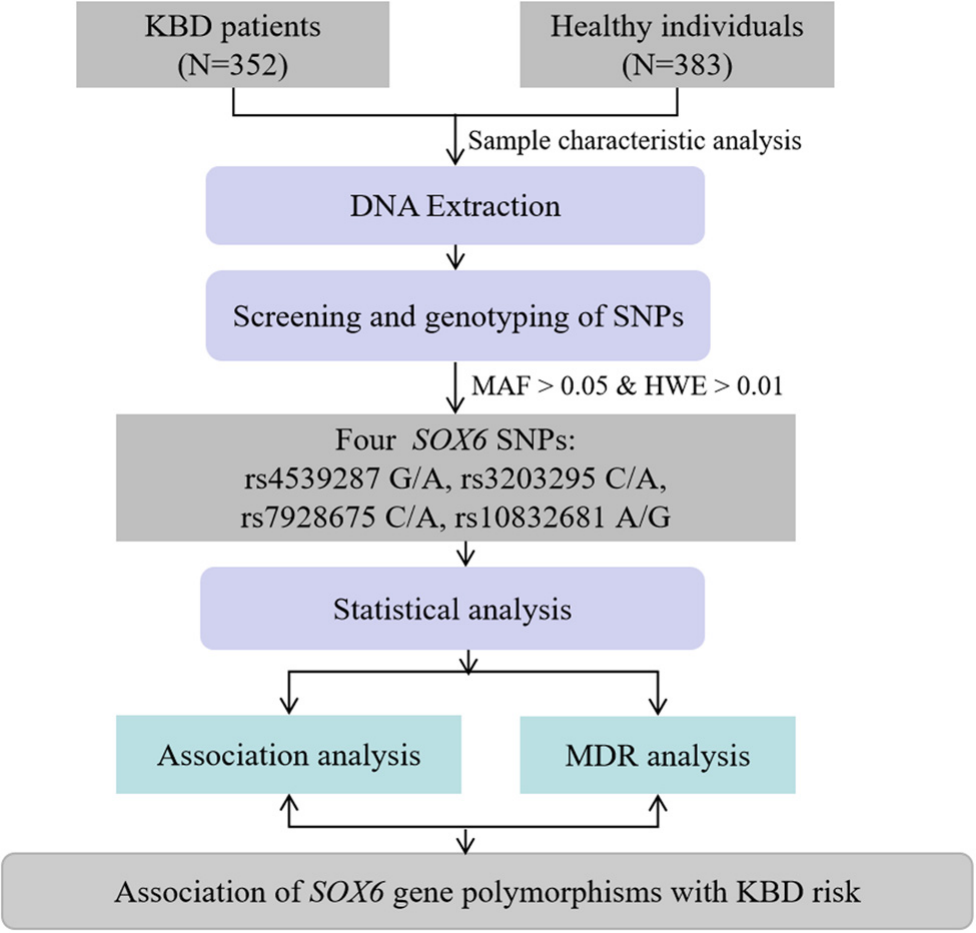
Flowchart of this study.

In overall and subgroup analyses, combined with genetic models (co-dominant, dominant, recessive, and additive models), logistic regression analysis was used to calculate odds ratios (ORs) and corresponding 95% confidence intervals (CIs) to further explore the correlation between candidate SNPs and KBD risk. Among them, we adjusted the ORs and 95% CIs through confounding factors. Subsequently, these positive results were verified through false positive report probability (FPRP) analysis. Among them, the FPRP value was less than 0.2 indicating that the correlation between *SOX6* SNPs and KBD risk was worth noting. We also explored the interaction between SNP and SNP based on the multi-factor dimensionality reduction (MDR) method. Figure 1 displays the detailed research process.

## 3 Results

### 3.1 Information about sample characteristics

This research involved 735 subjects, including 352 KBD patients (183 males and 169 females), with an average age of 65.32 ± 8.49 years; 383 healthy individuals (201 males and 182 females), with an average age of 64.19 ± 7.64 years. Among KBD patients, 195 patients (55.4%) experienced disease progression for more than 38 years, while 157 patients (44.6%) experienced disease progression for 38 years or less. Statistical analysis showed that there was no marked difference in age (*p* = 0.060), gender (*p* = 0.894), BMI (*p* = 0.692), and smoking (*p* = 0.974) between the case group and the control group, except for drinking (*p* = 0.024). More detailed information regarding the sample features is shown in Table 1.

**Table 1:** Sample characteristics information

Characteristics		Case *n* = 352	Control *n* = 383	*p*
Age (years)	Mean ± SD	65.32 ± 8.49	64.19 ± 7.64	**0.060** ^a^
	>65	166 (47.2%)	149 (38.9%)	
	≤65	186 (52.8%)	234 (61.1%)	
Gender	Male	183 (52%)	201 (52.5%)	**0.894** ^b^
	Female	169 (48%)	182 (47.5%)	
BMI	≥24	133 (37.8%)	173 (45.2%)	**0.692** ^a^
	<24	219 (62.2%)	210 (54.8%)	
Smoking	Yes	120 (34.1%)	131 (34.2%)	**0.974** ^b^
	No	232 (65.9%)	252 (65.8%)	
Drinking	Yes	44 (12.5%)	71 (18.5%)	0.024^b^
	No	308 (87.5%)	312 (81.5%)	
Course of disease (years)	>38	195 (55.4%)	—	−
	≤38	157 (44.6%)	−	
Number of affected joints	>5	220 (62.5%)	−	−
	≤5	132 (37.5%)	−	
Grade	2 vs 1	167 (47.4%)	69 (19.6%)	−
	3 vs 1	116 (33%)	69 (19.6%)	
	3 vs 2	116 (33%)	167 (47.4%)	
Hypertension	Non-hypertension	222 (63.1%)	−	−
	Hypertension	130 (36.9%)	−	
Diabetes	Non-diabetes	332 (94.3%)	−	−
	Diabetes	20 (5.7%)	−	

Notes: ^a^Independent samples *t*-test; ^b^Chi-squared test; *p* < 0.05 indicates statistical significance. Abbreviation: SD, standard deviation. Bold indicates no significant difference between cases and controls.

### 3.2 Information about *SOX6* SNPs

**Figure 2:**
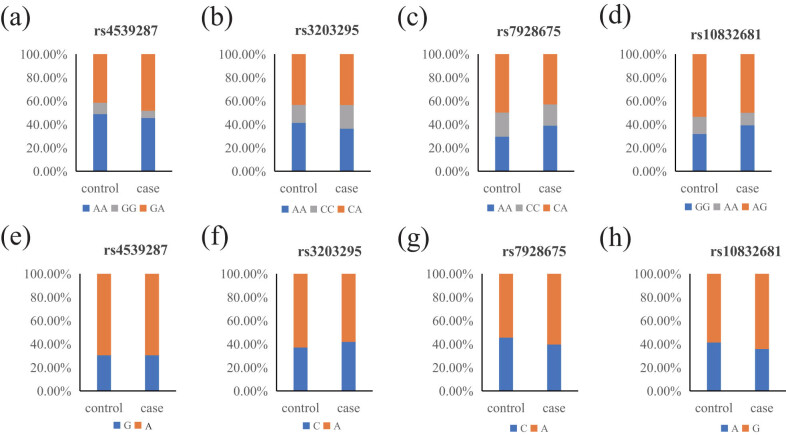
Genotype frequency and allele frequency of *SOX6* SNPs in KBD case and healthy control: (a) genotype frequency of rs4539287 between the two groups, (b) genotype frequency of rs3203295 between the two groups, (c) genotype frequency of rs7928675 between the two groups, (d) genotype frequency of rs10832681 between the two groups, (e) allele frequency of rs4539287 between the two groups, (f) allele frequency of rs3203295 between the two groups, (g) allele frequency of rs7928675 between the two groups, and (h) allele frequency of rs10832681 between the two groups.

The basic information of four candidate SNPs (rs4539287 G/A, rs3203295 C/A, rs7928675 C/A, rs10832681 A/G) is shown in Table 2. These SNPs are classified as untranslated regions (UTR) variants. The chi-square test demonstrated that these SNPs all comply with HWE (*p* > 0.05). The allele model analysis revealed that the minimum allele C of rs7928675 (OR = 0.78, 95% CI 0.64–0.97, *p* = 0.022) and the minimum allele A of rs10832681 (OR = 0.79, 95% CI 0.64–0.97, *p* = 0.028) could markedly reduce the KBD risk. In addition, the genotype frequency and allele frequency of the four candidate SNPs in the case group and control group are shown in Figure 2a–h and Table S2.

**Table 2:** Basic information of four candidate SNPs of *SOX6*

Gene	SNP ID	Function annotation	Chr: position	Alleles (A/B)	MAF		HWE (*p* value)	OR (95% CI)	*p* ^a^
					Cases	Controls			
*SOX6*	rs4539287	3′-UTR variant, 2KB upstream variant	11: 15,971,511	G/A	0.304	0.304	0.718	1.00 (0.80–1.25)	0.993
*SOX6*	rs3203295	5′-UTR variant, intron variant 2KB upstream variant	11: 16,740,086	C/A	0.419	0.371	0.188	1.22 (0.99–1.51)	0.058
*SOX6*	rs7928675	5′-UTR variant, intron variant, 2KB upstream variant	11: 16,740,259	C/A	0.396	0.456	1.000	0.78 (0.64–0.97)	0.022*
*SOX6*	rs10832681	3′-UTR variant	11: 16,778,008	A/G	0.358	0.414	0.058	0.79 (0.64–0.97)	0.028*

Notes: ^a^Chi-squared test; Bold and *p < 0.05 indicates statistical significance.

Abbreviations: SNP, single nucleotide polymorphisms; alleles (A/B), minor/major allele; MAF, minor allele frequency; HWE, Hardy–Weinberg equilibrium; OR, odds ratio; 95% CI, 95% confidence interval.

### 3.3 Association between *SOX6* SNPs and KBD risk (overall analysis)

**Figure 3:**
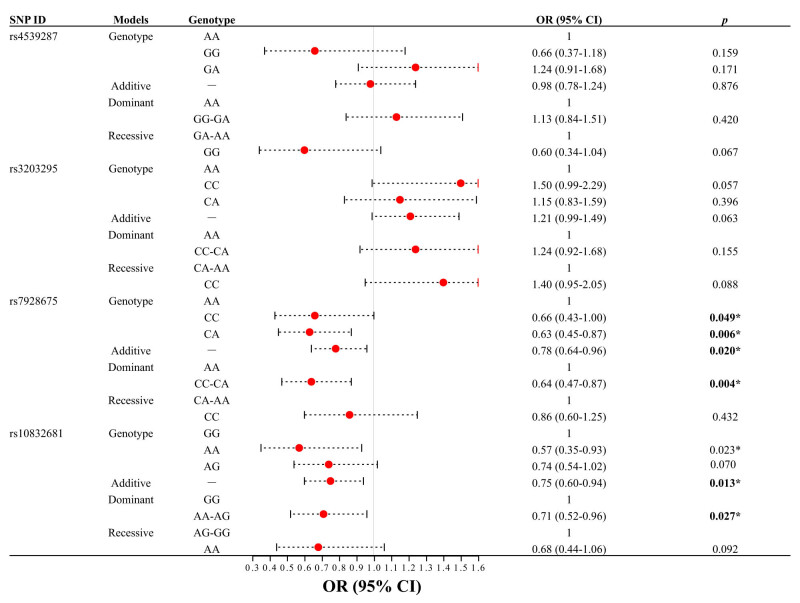
Association between four candidate SNPs of *SOX6* and KBD risk (overall analysis). *p < 0.05 indicates statistical significance.

Based on logistic regression analysis, this study conducted an overall correlation analysis between *SOX6* SNPs and KBD risk. The results revealed that (Figure 3) rs7928675 and rs10832681 were markedly correlated with a reduction in KBD risk. Specifically, rs7928675 markedly reduced the risk of KBD under homozygous (OR = 0.66, 95% CI 0.43–1.00, *p* = 0.049), heterozygote (OR = 0.63, 95% CI 0.45–0.87, *p* = 0.006), additive (OR = 0.78, 95% CI 0.64–0.96, *p* = 0.020), and dominant (OR = 0.64, 95% CI 0.47–0.87, *p* = 0.004) models. Similarly, rs10832681 also markedly reduced the risk of KBD under homozygous (OR = 0.57, 95% CI 0.35–0.93, *p =* 0.023), additive (OR = 0.75, 95% CI 0.60–0.94, *p =* 0.013), and dominant (OR = 0.71, 95% CI 0.52–0.96, *p =* 0.027) models. However, there was no significant correlation between rs4539287 and rs3203295 and KBD risk.

### 3.4 Association between *SOX6* SNPs and KBD risk (subgroup analysis)

**Figure 4:**
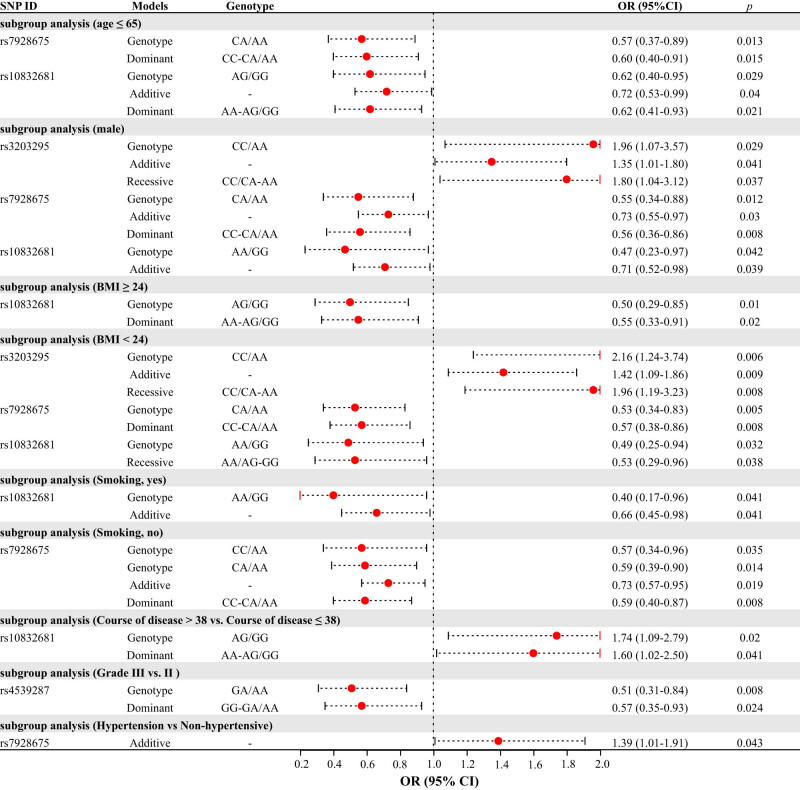
Association between four candidate SNPs of *SOX6* and KBD risk (subgroup analysis). Only significant results were shown. *p* < 0.05 indicates statistical significance.

In order to thoroughly explore the correlation between *SOX6* SNPs and KBD risk, this study performed subgroup analyses of age, gender, BMI, and smoking for all participants, as well as subgroup analyses of the course of disease, number of affected joints, grade (Ⅰ–Ⅲ), hypertensive complications, and diabetes complications for participants for KBD patients. The significant results of the subgroup association analysis between *SOX6* SNPs and KBD risk are shown in Figure 4.

Age-stratified analysis revealed that there was no correlation between *SOX6* SNPs and KBD risk in participants >65 years old (Table S3). However, for those aged 65 years or younger, rs7928675 under the heterozygote (OR = 0.57, 95% CI 0.37–0.89, *p* = 0.013) and dominant (OR = 0.60, 95% CI 0.40–0.91, *p* = 0.015) models, and rs10832681 under the heterozygote (OR = 0.62, 95% CI 0.40–0.95, *p* = 0.029), additive (OR = 0.72, 95% CI 0.53–0.99, *p* = 0.040), and dominant (OR = 0.62, 95% CI 0.41–0.93, *p* = 0.021) models could markedly reduce KBD risk.

Gender-stratified analysis showed that there was no marked correlation between *SOX6* SNPs and the risk of KBD among female participants (Table S4). However, for male participants, rs7928675 under the heterozygous (OR = 0.55, 95% CI 0.34–0.88, *p* = 0.012), additive (OR = 0.73, 95% CI 0.55–0.97, *p* = 0.030), dominant (OR = 0.56, 95% CI 0.36–0.86, *p* = 0.008), and rs10832681 under the homozygous (OR = 0.47, 95% CI 0.23–0.97, *p* = 0.042) and additive (OR = 0.71, 95% CI 0.52–0.98, *p* = 0.039) models could markedly decrease KBD risk. In addition, rs3203295 showed a significant increase in KBD risk in male participants under the homozygous (OR = 1.96, 95% CI 1.07–3.57, *p* = 0.029), additive (OR = 1.35, 95% CI 1.01–1.80, *p* = 0.041), and received (OR = 1.80, 95% CI 1.04–3.12, *p* = 0.037) models.

BMI-stratified analysis showed that rs10832681 (Table S5) was related to a decreased KBD risk in participants with a BMI of 24 or higher under the heterozygote (OR = 0.50, 95% CI 0.29–0.85, *p* = 0.010) and dominant (OR = 0.55, 95% CI 0.33–0.91, *p* = 0.020) models. In those with BMI below 24, except for rs4539287, there was a certain correlation between the other three *SOX6* SNPs and KBD risk. Of these three, rs3203295 could markedly increase the risk of KBD under the homozygous (OR = 2.16, 95% CI 1.24–3.74, *p* = 0.006), additive (OR = 1.42, 95% CI 1.09–1.86, *p* = 0.009), and recessive (OR = 1.96, 95% CI 1.19–3.23, *p* = 0.008) models. While rs7928675 under the heterozygote (OR = 0.53, 95% CI 0.34–0.83, *p* = 0.005) and dominant (OR = 0.57, 95% CI 0.38–0.86, *p* = 0.008) models, and rs10832681 under the homozygous (OR = 0.49, 95% CI 0.25–0.94, *p* = 0.032) and recessive (OR = 0.53, 95% CI 0.29–0.96, *p* = 0.038) models could markedly reduce the risk of KBD.

Smoking-stratified analysis found that (Table S6) rs7928675 markedly reduced the risk of KBD in non-smokers under the homozygous (OR = 0.57, 95% CI 0.34–0.96, *p* = 0.035), heterozygote (OR = 0.59, 95% CI 0.39–0.90, *p* = 0.014), additive (OR = 0.73, 95% CI 0.57–0.95, *p* = 0.019), and dominant (OR = 0.59, 95% CI 0.40–0.87, *p* = 0.008) models. And rs10832681 could markedly reduce the KBD risk in smokers under the homozygous (OR = 0.40, 95% CI 0.17–0.96, *p* = 0.041) and additive (OR = 0.66, 95% CI 0.45–0.98, *p* = 0.041) models.

The course of disease stratified analysis revealed that (Table S7) only rs10832681 markedly increased the disease risk in KBD patients with the course of disease longer than 38 years.

The number of affected joints stratified analysis showed that (Table S8) the correlation between *SOX6* SNPs and KBD risk was not related to the number of damaged joints in patients.

Based on the severity of joint lesions, this study classified KBD patients into Ⅰ–Ⅲ grades and investigated the correlation between *SOX6* SNPs and KBD risk among different grades. The results showed that (Table S9), with grade Ⅱ as the control group, rs4539287 could markedly decrease the KBD risk in grade Ⅲ patients under the heterozygote (OR = 0.51, 95% CI 0.31–0.84, *p* = 0.008) and dominant (OR = 0.57, 95% CI 0.35–0.93, *p* = 0.024) models.

Hypertensive and diabetes complications stratified analyses showed that (Tables S10 and S11) the link between *SOX6* SNPs and the KBD risk was not markedly impacted by whether or not the patient had hypertension or diabetes. Only rs7928675 had a marked effect on increasing the KBD risk in hypertensive patients under the additive (OR = 0.76, 95% CI 0.59–0.97, *p* = 0.025) model.

### 3.5 FPRP analysis

Based on the FPRP analysis, we proceeded to conduct a reliability analysis on the positive results mentioned above (Table S12). In the overall analysis and subgroup analysis of males, BMI < 24, and non-smoking, the link between rs10832681 and KBD risk under the homozygous model should not be a concern. In the overall analysis and subgroup analysis of non-smoking, the link between rs7928675 and KBD risk under the homozygous model should not be of concern. Additionally, the correlation between rs3203295 and KBD risk under homozygous and recessive models in males should not be of concern. It is noteworthy that other positive results had FPRP values less than 0.2, showing a significant correlation between *SOX6* SNPs and KBD risk, which warrants further in-depth analysis.

### 3.6 SNP–SNP interaction analysis

**Figure 5:**
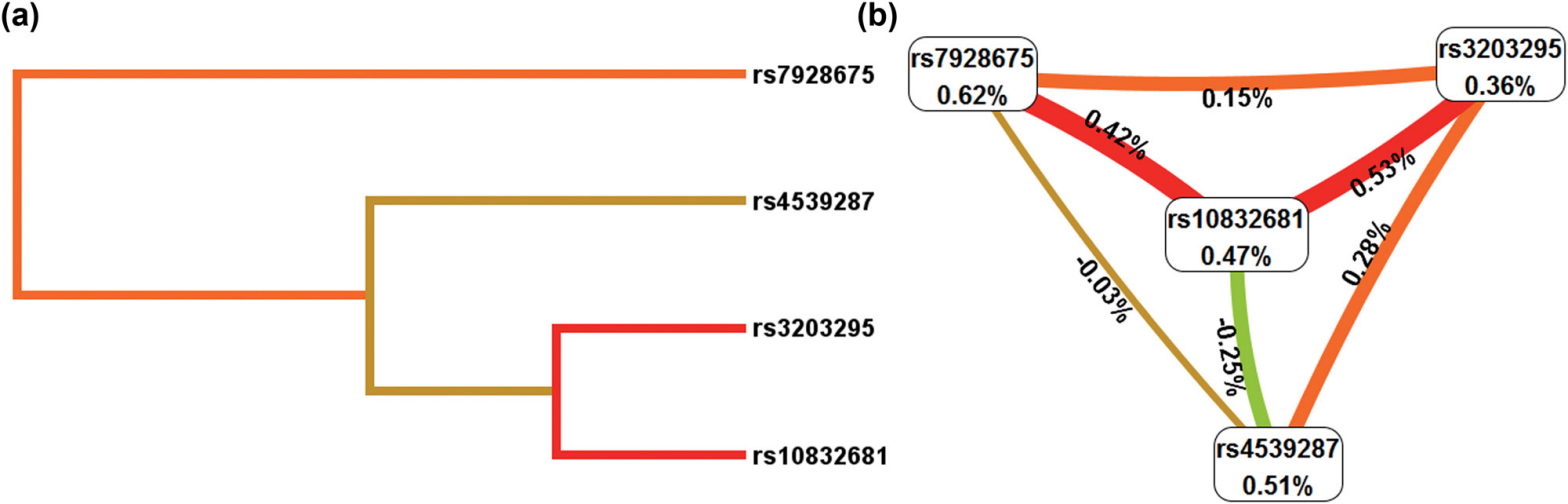
Dendrogram (a) and circle graph (b) of SNP–SNP interaction among *SOX6* SNPs by MDR method. The numerical values on the lines represent the strength of the interaction.

This study evaluated the correlation between SNP–SNP in *SOX6* polymorphism using the MDR method. The results revealed a significant interaction between rs3203295 and rs10832681 (Figure 5a and b). Meanwhile, the 4-site combination model, which includes rs4539287, rs3203295, rs7928675, and rs10832681, was the most effective model for predicting KBD risk, with a testing balanced accuracy of 0.5355 and cross-validation consistency (CVC) of 10/10 (Table 3).

**Table 3:** SNP–SNP interactions in KBD risk based on MDR analysis

Models	Training Bal. Acc	Testing Bal. Acc	OR (95% CI)	*p*	CVC
rs7928675	0.5456	0.5014	1.48 (1.08–2.02)	0.014	7/10
rs4539287, rs3203295	0.5677	0.5071	1.66 (1.23–2.25)	0.001	6/10
rs4539287, rs3203295, rs7928675	0.6002	0.5185	2.19 (1.62–2.97)	<0.0001	7/10
rs4539287, rs3203295, rs7928675, rs10832681	0.6302	0.5355	2.96 (2.16–4.06)	<0.0001	10/10

Abbreviations: MDR, multifactor dimensionality reduction; Bal. Acc, balanced accuracy; CVC, cross-validation consistency.

## 4 Discussion

This study aims to analyze the potential correlation between four specific *SOX6* SNPs (rs4539287, rs3203295, rs7928675, and rs10832681) and KBD risk among 735 participants, to screen for more genetic variations related to the occurrence and progression of KBD. Overall analysis revealed a significant correlation between rs7928675 and rs10832681 and a decrease in KBD risk. Further subgroup analyses indicated that these two SNPs also have a significant protective effect on KBD risk among participants who are aged 65 years or younger, males, and non-smokers.

The *SOX6* gene plays a crucial role as a transcription factor in the development and formation of cartilage [19]. Yang et al. conducted a fine-mapping association analysis and found that there are numerous *SOX6* SNPs related to hip bone mineral density in both Chinese and Caucasian populations, emphasizing the importance of *SOX6* gene variants in affecting changes in bone mineral density [20]. A cross-sectional study by Correa-Rodríguez et al. revealed that *SOX6* rs7117858 can affect the fat free mass and quantitative ultra sound characteristics of young people, indicating the significance of *SOX6* variants in obesity and osteoporosis-related phenotypes during early adulthood [17]. Furthermore, a case–control study by Wu et al. revealed a relationship between *SOX6* mutations and adolescent idiopathic scoliosis in the Han Chinese population [21]. These studies suggested a potential connection between *SOX6* gene variants and bone-related diseases, providing important theoretical support for the first in-depth exploration of the link between *SOX6* SNPs and KBD risk in this study.

KBD is more common in children and adolescents, particularly those between the ages of 5–15 years [22]. Through subgroup analysis, it has been observed that rs7928675 and rs10832681 significantly reduced the risk of KBD in younger individuals (age ≤65 years), suggesting that these SNPs may be key in KBD among young people. Meanwhile, this study also found that these SNPs have a certain protective effect in males, and non-smokers, suggesting that gender may be crucial in the association between *SOX6* SNPs and KBD risk and that the adverse effects of smoking on the development of KBD may interfere with the protective effect of these SNPs. It is essential to note that although these findings provide insight into the link between *SOX6* SNPs and KBD risk, more research is still necessary to verify the potential connection between factors such as gender, smoking, and KBD.

Genetic mutations can occur in coding and non-coding regions of the genome. Previous research has shown that both of them can impact gene expression, potentially leading to the development of diseases [23]. Du et al. pointed out that the polymorphism of *GPx4* and the decrease in its mRNA expression level may be related to the development of KBD in the Chinese population [24]. Similarly, when Sun et al. investigated the correlation between the *SEPP1* variant and the risk of KBD, they also tested the expression of *SEPP1* gene mRNA in patients to analyze the potential link between *SEPP1* SNPs and the pathogenesis of KBD [25]. In this study, we found that rs7928675 and rs10832681 located in the non-coding region of the *SOX6* were significantly associated with a reduction in KBD risk. Previous studies have reported that *SOX6* plays an important role in cartilage formation and bone development, and its inactivation may affect the differentiation of chondrocytes and neuronal cells, leading to the occurrence of bone-related diseases [14,15]. Considering that both rs7928675 and rs10832681 are located in non-coding regions, it may affect the binding ability of *SOX6* regulatory elements or the role of regulatory factors, which may lead to changes in the expression level of *SOX6*. Therefore, we speculated that the specific alleles of these two SNPs may enhance the interaction between *SOX6* regulatory elements and transcription factors, thereby increasing the expression of *SOX6* and reducing the risk of KBD. However, this hypothesis needs to be further validated through experimental methods such as gene expression analysis and chromatin conformation.

It is undeniable that this study has some limitations. First, the sample size was relatively small and the majority of participants are local residents of Changwu County, with only a small number from other cities or provinces, indicating an uneven geographical distribution of the participants in this study. Considering that participants from this specific region may have specific genetic polymorphisms, in future related studies, we will expand the sample size and include participants from different regions to further validate the association between *SOX6* SNPs and KBD risk in the Chinese Han population. Second, due to significant differences between the case group and the control group in drinking, this study did not conduct a drinking-standardized analysis regarding the association between *SOX6* SNPs and KBD risk. In subsequent studies, we will further expand the sample size to ensure that the case group matches the control group, and then proceed with the drinking-standardized analysis of the correlation between *SOX6* SNPs and KBD risk to obtain reliable results related to drinking. Additionally, this study found a marked correlation between the two candidate SNPs of *SOX6* and the reduction of KBD risk, which needs to be further validated through relevant experiments.

## 5 Conclusions

This study revealed a significant correlation between *SOX6* variants (rs7928675 and rs10832681) and a reduction in the KBD risk among the Chinese Han population, providing a new direction for the prevention, diagnosis, and treatment of KBD.

## Abbreviations

CIs: confidence intervals
CVC: cross-validation consistency
FFM: fat free mass
FPRP: false positive report probability
*GPx4*
: glutathione peroxidase 4
HWE: Hardy–Weinberg equilibrium
KBD: Kashin-Beck disease
MAF: minor allele frequency
MDR: multi-factor dimensionality reduction
ORs: odds ratios
QUS: quantitative ultra sound
Se: selenium
*SEPP1*
: selenoprotein P
SNPs: single nucleotide polymorphisms
*SOX6*
: SRY-box transcription factor 6
TOLCAS: Tolchin-Le Caignec syndrome

## Supplementary Material

Supplementary material

## References

[1] Shao Q, Yang H. Severe deformity in long-term Kaschin–Beck disease. Rheumatology. 2022;61(4):1732. 10.1093/rheumatology/keab486. 34128968

[2] Cao J, Li S, Shi Z, Yue Y, Sun J, Chen J, et al. Articular cartilage metabolism in patients with Kashin–Beck disease: an endemic osteoarthropathy in China. Osteoarthr Cartil. 2008;16(6):680–8. 10.1016/j.joca.2007.09.002. 17945513

[3] Tan J, Zhu W, Wang W, Li R, Hou S, Wang D, et al. Selenium in soil and endemic diseases in China. Sci Total Environ. 2002;284(1–3):227–35. 10.1016/s0048-9697(01)00889-0 11846167

[4] Lv Y, Yu T, Yang Z, Zhao W, Zhang M, Wang Q. Constraint on selenium bioavailability caused by its geochemical behavior in typical Kaschin–Beck disease areas in Aba, Sichuan Province of China. Sci Total Environ. 2014;493:737–49. 10.1016/j.scitotenv.2014.06.050. 24995640

[5] Commission NHaFP. China health and family planning statistical yearbook 2016. China: Beijing Union Medical University Press; 2016.

[6] Shi X, Lv A, Ma J, Zhang F, Wen Y, Zhang Z, et al. Investigation of MMP-1 genetic polymorphisms and protein expression and their effects on the risk of Kashin-Beck disease in the northwest Chinese Han population. J Orthopaedic Surg Res. 2016;11(1):64. 10.1186/s13018-016-0398-6. PMC4888510 27245218

[7] Bai Y, Shi X, Zhang F, Lv A, Wen Y, Guo X. COL9A1 gene polymorphism is associated with Kashin-Beck disease in a Northwest Chinese Han population. PLoS One. 2015;10(3):e0120365. 10.1371/journal.pone.0120365. PMC4361735 25774918

[8] He X, Bai M, Liu M, Wang L, He Y, Rong H, et al. Genetic variants in the ITPR2 gene are associated with Kashin‐Beck disease in Tibetan. Mol Genet Genomic Med. 2019;7(7):e00715. 10.1002/mgg3.715. PMC6625103 31066235

[9] Zhang F, Guo X, Zhang Y, Wen Y, Wang W, Wang S, et al. Genome-wide copy number variation study and gene expression analysis identify ABI3BP as a susceptibility gene for Kashin-Beck disease. Hum Genet. 2014;133(6):793–9. 10.1007/s00439-014-1418-4 24442417

[10] Wu C, Wen Y, Guo X, Yang T, Shen H, Chen X, et al. Genetic association, mRNA and protein expression analysis identify ATG4C as a susceptibility gene for Kashin–Beck disease. Osteoarthr Cartil. 2017;25(2):281–6. 10.1016/j.joca.2016.09.019. 27742532

[11] Yu FF, Sun L, Zhou GY, Ping ZG, Guo X, Ba Y. Meta-analysis of association studies of selenoprotein gene polymorphism and Kashin-Beck disease: an updated systematic review. Biol Trace Elem Res. 2022;200:543–50. 10.1007/s12011-021-02705-2 33844169

[12] Akiyama H, Chaboissier M, Martin JF, Schedl A, de Crombrugghe B. The transcription factor Sox9 has essential roles in successive steps of the chondrocyte differentiation pathway and is required for expression of Sox5 and Sox6. Genes Dev. 2002;16(21):2813–28. 10.1101/gad.1017802. PMC187468 12414734

[13] Liu C, Lefebvre V. The transcription factors SOX9 and SOX5/SOX6 cooperate genome-wide through super-enhancers to drive chondrogenesis. Nucleic Acids Res. 2015;43(17):8183–203. 10.1093/nar/gkv688. PMC4787819 26150426

[14] Smits P, Li P, Mandel J, Zhang Z, Deng JM, Behringer RR, et al. The transcription factors L-Sox5 and Sox6 are essential for cartilage formation. Dev Cell. 2001;1(2):277–90. 10.1016/s1534-5807(01)00003-x 11702786

[15] Dy P, Smits P, Silvester A, Penzo-Méndez A, Dumitriu B, Han Y, et al. Synovial joint morphogenesis requires the chondrogenic action of Sox5 and Sox6 in growth plate and articular cartilage. Dev Biol. 2010;341(2):346–59. 10.1016/j.ydbio.2010.02.024. PMC2862098 20206616

[16] Tolchin D, Yeager JP, Prasad P, Dorrani N, Russi AS, Martinez-Agosto JA, et al. De dovo SOX6 variants cause a neurodevelopmental syndrome associated with ADHD, craniosynostosis, and osteochondromas. Am J Hum Genet. 2020;106(6):830–45. 10.1016/j.ajhg.2020.04.015. PMC7273536 32442410

[17] Correa-Rodríguez M, Schmidt-RioValle J, Rueda-Medina B. SOX6 rs7117858 polymorphism is associated with osteoporosis and obesity-related phenotypes. Eur J Clin Investig. 2018;48(10):e13011. 10.1111/eci.13011. 30062780

[18] Hsu T, Tantoh DM, Chou Y, Hsu S, Ho C, Lung C, et al. Association between osteoporosis and menopause in relation to SOX6 rs297325 variant in Taiwanese women. Menopause. 2020;27(8):887–92. 10.1097/gme.0000000000001544. PMC7386873 32187136

[19] Nishimura R, Hata K, Takahata Y, Murakami T, Nakamura E, Yagi H. Regulation of cartilage development and diseases by transcription factors. J Bone Metab. 2017;24(3):147–53. 10.11005/jbm.2017.24.3.147. PMC5613019 28955690

[20] Yang TL, Guo Y, Liu YJ, Shen H, Liu YZ, Lei SF, et al. Genetic variants in the SOX6 gene are associated with bone mineral density in both Caucasian and Chinese populations. Osteoporos Int. 2011;23(2):781–7. 10.1007/s00198-011-1626-x. PMC4171834 21625884

[21] Wu Z, Wang Y, Dai Z, Qiu Y, Xu L, Zhu Z. Genetic variants of ABO and SOX6 are associated with adolescent idiopathic scoliosis in Chinese Han Population. Spine. 2019;44(18):E1063–7. 10.1097/brs.0000000000003062. 30994600

[22] Sun LY, Meng FG, Li Q, Zhao ZJ, He CZ, Wang SP, et al. Effects of the consumption of rice from non-KBD areas and selenium supplementation on the prevention and treatment of paediatric Kaschin–Beck disease: an epidemiological intervention trial in the Qinghai Province. Osteoarthr Cartil. 2014;22(12):2033–40. 10.1016/j.joca.2014.09.013. 25252032

[23] Shastry BS. SNPs: impact on gene function and phenotype. Methods Mol Biol. 2009;578:3–22. 10.1007/978-1-60327-411-1_1. 19768584

[24] Du XH, Dai XX, Xia SR, Zou XZ, Sun WY, Mo XY, et al. SNP and mRNA expression for glutathione peroxidase 4 in Kashin-Beck disease. Br J Nutr. 2011;107(2):164–9. 10.1017/s0007114511002704. 21733339

[25] Sun W, Wang X, Zou XZ, Song R, Du X, Hu J, et al. Selenoprotein P gene r25191g/a polymorphism and quantification of selenoprotein P mRNA level in patients with Kashin-Beck disease. Br J Nutr. 2010;104(9):1283–7. 10.1017/s0007114510002199. 20565998

