# Supplementary material for: Association of SOX6 gene polymorphisms with Kashin-Beck disease risk in the Chinese Han population
Source: Open Med (Wars). 2024 Jan 6;19(1):20230883. doi: 10.1515/med-2023-0883 (PMC10775412; doi:10.1515/med-2023-0883)
Supplement: Supplementary material [file med-2023-0883-sm.pdf]

Supplementary material

Table S1: Primer sequence information about four candidate SNPs of SOX6

SNP_ID	Second PCR primer	First PCR primer	UEP_DIR	UEP_SEQ
rs4539287	ACGTTGGATGGTGACCTCAAGTCTTTTGC	ACGTTGGATGGAAATGCCACTGATGCAGAC	R	cattgTCCCCTAATTCAGTTCACAAG
rs3203295	ACGTTGGATGCTGACAAAACCTGAATTACC	ACGTTGGATGTGAATGGGCACCTAGAGAAG	R	ccccaATATTAACAAGATCTAAAA
rs7928675	ACGTTGGATGCCTCTTCCGAAGCTGTAAAC	ACGTTGGATGGATATCAGACACCTGTTTGG	R	gaatgTCATATCACCTGGAGTTT
rs10832681	ACGTTGGATGAAAATGAAAGCAGGCTGGG	ACGTTGGATGTCAGGTGATTCATCCGCTTC	F	aAGGCTGGGCGTGGTGGCTCAC

Abbreviations: UEP_SEQ: unextended mini-sequencing primer.

Table S2: The genotype frequency and allele frequency of *SOX6* SNPs in KBD case and healthy control

SNP ID	Genotype / Allele	Control	Case
rs4539287	AA	187 (48.82%)	160 (45.45%)
	GG	37 (9.67%)	22 (6.25%)
	GA	159 (41.51%)	170 (48.30%)
	G	233 (30.42%)	214 (30.40%)
	A	533 (69.58%)	490 (69.60%)
rs3203295	AA	158 (41.25%)	128 (36.36%)
	CC	59 (15.40%)	71 (20.17%)
	CA	166 (43.35%)	153 (43.47%)
	C	284 (37.08%)	295 (41.90%)
	A	482 (62.92%)	409 (58.10%)
rs7928675	AA	113 (29.50%)	137 (38.92%)
	CC	79 (20.63%)	64 (18.18%)
	CA	191 (49.87%)	151 (42.90%)
	C	349 (45.56%)	279 (39.63%)
	A	417 (54.44%)	425 (60.37%)
rs10832681	GG	122 (31.85%)	138 (39.20%)
	AA	56 (14.62%)	38 (10.80%)
	AG	205 (53.53%)	176 (50%)
	A	317 (41.38%)	252 (35.80%)
	G	449 (58.62%)	452 (64.20%)

Table S3: Association between four candidate SNPs of *SOX6* and KBD risk (age-stratified analysis)

SNP ID	Models	Genotype	Age, years			
			**OR (95% CI)** ≤ **65 (N = 420)**	***p***	**OR (95% CI)** > **65 (N = 315)**	***p***
rs4539287	Genotype	AA	1.00		1.00	
		GG	0.63 (0.28–1.42)	0.268	0.64 (0.28–1.50)	0.307
		GA	1.22 (0.81–1.82)	0.339	1.25 (0.78–2.01)	0.361
	Additive	-	0.98 (0.71–1.33)	0.876	0.96 (0.67–1.38)	0.841
	Dominant	AA	1.00		1.00	
		GG-GA	1.12 (0.76–1.65)	0.583	1.12 (0.71–1.77)	0.618
	Recessive	GA-AA	1.00		1.00	
		GG	0.57 (0.26–1.25)	0.164	0.58 (0.26–1.30)	0.183
rs3203295	Genotype	AA	1.00		1.00	
		CC	1.37 (0.79–2.39)	0.267	1.53 (0.78–3.00)	0.212
		CA	1.26 (0.82–1.95)	0.296	0.92 (0.56–1.51)	0.733
	Additive	-	1.18 (0.90–1.55)	0.219	1.17 (0.85–1.61)	0.339
	Dominant	AA	1.00		1.00	
		CC-CA	1.29 (0.86–1.94)	0.213	1.06 (0.66–1.68)	0.820
	Recessive	CA-AA	1.00		1.00	
		CC	1.21 (0.73–2.01)	0.457	1.61 (0.87–2.97)	0.131
rs7928675	Genotype	AA	1.00		1.00	
		CC	0.69 (0.39–1.20)	0.184	0.69 (0.36–1.33)	0.270
		CA	0.57 (0.37–0.89)	0.013*	0.75 (0.44–1.27)	0.288
	Additive	-	0.78 (0.59–1.03)	0.077	0.82 (0.60–1.14)	0.243
	Dominant	AA	1.00		1.00	
		CC-CA	0.60 (0.40–0.91)	0.015*	0.73 (0.45–1.21)	0.224
	Recessive	CA-AA	1.00		1.00	
		CC	0.93 (0.56–1.54)	0.776	0.83 (0.47–1.45)	0.512
rs10832681	Genotype	GG	1.00		1.00	
		AA	0.60 (0.31–1.15)	0.125	0.58 (0.27–1.21)	0.147
		AG	0.62 (0.40–0.95)	0.029*	1.00 (0.61–1.64)	0.992
	Additive	-	0.72 (0.53–0.99)	0.040*	0.82 (0.58–1.16)	0.257
	Dominant	GG	1.00		1.00	
		AA-AG	0.62 (0.41-0.93)	0.021*	0.90 (0.56–1.44)	0.659
	Recessive	AG-GG	1.00		1.00	
		AA	0.80 (0.44–1.46)	0.472	0.58 (0.29–1.14)	0.113

Notes: *$p < 0.05$ indicates statistical significance.

Abbreviations: SNP, single nucleotide polymorphism; OR, odds ratio; CI, confidence interval.

Table S4: Association between four candidate SNPs of *SOX6* and KBD risk (gender-stratified analysis)

SNP ID	Models	Genotype	Gender			
			OR (95% CI) Female (N = 351)	*p*	OR (95% CI) Male (N = 384)	*p*
rs4539287	Genotype	AA	1.00		1.00	
		GG	0.61 (0.27–1.37)	0.231	0.81 (0.35–1.90)	0.635
		GA	1.39 (0.89–2.19)	0.150	1.16 (0.76–1.77)	0.493
	Additive	-	0.99 (0.71–1.37)	0.935	1.02 (0.73–1.43)	0.899
	Dominant	AA	1.00		1.00	
		GG-GA	1.21 (0.79–1.86)	0.383	1.11 (0.74–1.67)	0.623
	Recessive	GA-AA	1.00		1.00	
		GG	0.52 (0.24–1.13)	0.098	0.76 (0.33–1.72)	0.507
rs3203295	Genotype	AA	1.00		1.00	
		CC	1.24 (0.67–2.28)	0.494	1.96 (1.07–3.57)	**0.029***
		CA	1.11 (0.69–1.78)	0.678	1.17 (0.74–1.83)	0.503
	Additive	-	1.11 (0.83–1.49)	0.484	1.35 (1.01–1.80)	**0.041***
	Dominant	AA	1.00		1.00	
		CC-CA	1.14 (0.74–1.78)	0.549	1.34 (0.88–2.05)	0.169
	Recessive	CA-AA	1.00		1.00	
		CC	1.18 (0.67–2.06)	0.572	1.80 (1.04–3.12)	**0.037***
rs7928675	Genotype	AA	1.00		1.00	
		CC	0.68 (0.36–1.27)	0.229	0.58 (0.32–1.03)	0.063
		CA	0.66 (0.40–1.07)	0.092	0.55 (0.34–0.88)	**0.012***
	Additive	-	0.80 (0.59–1.09)	0.155	0.73 (0.55–0.97)	**0.030***
	Dominant	AA	1.00		1.00	
		CC-CA	0.66 (0.42–1.05)	0.081	0.56 (0.36–0.86)	**0.008***
	Recessive	CA-AA	1.00		1.00	
		CC	0.88 (0.50–1.52)	0.639	0.81 (0.49–1.35)	0.425
rs10832681	Genotype	GG	1.00		1.00	
		AA	0.60 (0.30–1.20)	0.149	0.47 (0.23–0.97)	**0.042***
		AG	0.67 (0.41–1.08)	0.101	0.76 (0.49–1.18)	0.228
	Additive	-	0.75 (0.54–1.05)	0.091	0.71 (0.52–0.98)	**0.039***
	Dominant	GG	1.00		1.00	
		AA-AG	0.65 (0.41–1.04)	0.073	0.70 (0.46–1.07)	0.100
	Recessive	AG-GG	1.00		1.00	
		AA	0.78 (0.42–1.44)	0.428	0.55 (0.28–1.09)	0.085

Notes: *$p < 0.05$ indicates statistical significance.

Abbreviations: SNP, single nucleotide polymorphism; OR, odds ratio; CI, confidence interval.

Table S5: Association between four candidate SNPs of *SOX6* and KBD risk (BMI-stratified analysis)

SNP ID	Models	Genotype	BMI			
			OR (95% CI) ≥ 24 (N = 306)	*p*	OR (95% CI) < 24 (*N* = 429)	*p*
rs4539287	Genotype	AA	1.00		1.00	
		GG	0.41 (0.12–1.35)	0.142	0.81 (0.40–1.61)	0.541
		GA	1.39 (0.85–2.27)	0.191	1.16 (0.77–1.75)	0.473
	Additive	-	0.99 (0.67–1.47)	0.971	0.99 (0.74–1.34)	0.953
	Dominant	AA	1.00		1.00	
		GG-GA	1.22 (0.76–1.97)	0.410	1.09 (0.74–1.61)	0.672
	Recessive	GA-AA	1.00		1.00	
		GG	0.35 (0.11–1.12)	0.077	0.75 (0.39–1.46)	0.396
rs3203295	Genotype	AA	1.00		1.00	
		CC	0.76 (0.37–1.59)	0.474	2.16 (1.24–3.74)	0.006*
		CA	1.13 (0.68–1.90)	0.631	1.20 (0.78–1.85)	0.417
	Additive	-	0.93 (0.66–1.31)	0.682	1.42 (1.09–1.86)	0.009*
	Dominant	AA	1.00		1.00	
		CC-CA	1.03 (0.64–1.68)	0.898	1.44 (0.97–2.15)	0.074
	Recessive	CA-AA	1.00		1.00	
		CC	0.71 (0.36–1.41)	0.331	1.96 (1.19–3.23)	0.008*
rs7928675	Genotype	AA	1.00		1.00	
		CC	0.63 (0.32–1.25)	0.186	0.66 (0.38–1.16)	0.150
		CA	0.80 (0.47–1.38)	0.421	0.53 (0.34–0.83)	0.005*
	Additive	-	0.80 (0.57–1.11)	0.180	0.77 (0.59–1.01)	0.061
	Dominant	AA	1.00		1.00	
		CC-CA	0.75 (0.45–1.24)	0.257	0.57 (0.38–0.86)	0.008*
	Recessive	CA-AA	1.00		1.00	
		CC	0.72 (0.39–1.32)	0.284	0.96 (0.58–1.56)	0.855
rs10832681	Genotype	GG	1.00		1.00	
		AA	0.78 (0.36-1.71)	0.539	0.49 (0.25–0.94)	0.032*
		AG	0.50 (0.29–0.85)	0.010*	0.88 (0.58–1.34)	0.548
	Additive	-	0.76 (0.52–1.10)	0.146	0.75 (0.55–1.01)	0.057
	Dominant	GG	1.00		1.00	
		AA-AG	0.55 (0.33–0.91)	0.020*	0.79 (0.53–1.18)	0.251
	Recessive	AG-GG	1.00		1.00	
		AA	1.19 (0.59–2.41)	0.629	0.53 (0.29–0.96)	0.038*

Notes: *$p < 0.05$ indicates statistical significance.

Abbreviations: SNP, single nucleotide polymorphism; OR, odds ratio; CI, confidence interval.

Table S6: Association between four candidate SNPs of *SOX6* and KBD risk (Smoking-stratified analysis)

SNP ID	Models	Genotype	Smoking			
			**OR (95% CI)** **Yes (*N* = 251)**	***p***	**OR (95% CI)** **No (*N* = 484)**	***p***
rs4539287	Genotype	AA	1.00		1.00	
		GG	0.51 (0.16–1.60)	0.247	0.74 (0.38–1.45)	0.377
		GA	1.08 (0.64–1.82)	0.785	1.40 (0.96–2.06)	0.084
	Additive	-	0.89 (0.58–1.35)	0.583	1.05 (0.79–1.39)	0.755
	Dominant	AA	1.00		1.00	
		GG-GA	0.99 (0.59–1.64)	0.955	1.26 (0.87–1.82)	0.216
	Recessive	GA-AA	1.00		1.00	
		GG	0.49 (0.16–1.50)	0.212	0.62 (0.33–1.19)	0.152
rs3203295	Genotype	AA	1.00		1.00	
		CC	2.04 (0.94–4.43)	0.070	1.36 (0.81–2.27)	0.243
		CA	1.14 (0.65–2.00)	0.661	1.15 (0.77–1.72)	0.498
	Additive	-	1.37 (0.94–1.97)	0.097	1.16 (0.91–1.49)	0.235
	Dominant	AA	1.00		1.00	
		CC-CA	1.32 (0.78–2.25)	0.306	1.21 (0.83–1.75)	0.316
	Recessive	CA-AA	1.00		1.00	
		CC	1.90 (0.94–3.85)	0.073	1.27 (0.79–2.03)	0.328
rs7928675	Genotype	AA	1.00		1.00	
		CC	0.77 (0.37–1.63)	0.499	0.57 (0.34–0.96)	0.035*
		CA	0.60 (0.34–1.07)	0.081	0.59 (0.39–0.90)	0.014*
	Additive	-	0.83 (0.58–1.19)	0.312	0.73 (0.57–0.95)	0.019*
	Dominant	AA	1.00		1.00	
		CC-CA	0.64 (0.37–1.11)	0.110	0.59 (0.40–0.87)	0.008*
	Recessive	CA-AA	1.00		1.00	
		CC	1.04 (0.54–2.02)	0.906	0.78 (0.49–1.23)	0.279
rs10832681	Genotype	GG	1.00		1.00	
		AA	0.40 (0.17–0.96)	0.041*	0.65 (0.36–1.18)	0.155
		AG	0.73 (0.42–1.27)	0.269	0.76 (0.51–1.14)	0.180
	Additive	-	0.66 (0.45–0.98)	0.041*	0.79 (0.60–1.05)	0.106
	Dominant	GG	1.00		1.00	
		AA-AG	0.65 (0.38–1.11)	0.113	0.74 (0.50–1.08)	0.121
	Recessive	AG-GG	1.00		1.00	
		AA	0.48 (0.21–1.08)	0.077	0.77 (0.45–1.32)	0.342

Notes: *$p < 0.05$ indicates statistical significance.

Abbreviations: SNP, single nucleotide polymorphism; OR, odds ratio; CI, confidence interval.

Table S7: Association between four candidate SNPs of *SOX6* and KBD risk (Course of disease stratified analysis)

SNP ID	Models	Genotype	Course of disease > 38 vs. Course of disease ≤ 38	
			OR (95% CI)	*p*
rs4539287	Genotype	AA	1.00	
		GG	0.72 (0.28–1.83)	0.490
		GA	0.96 (0.61–1.50)	0.850
	Additive	-	0.90 (0.63–1.30)	0.586
	Dominant	AA	1.00	
		GG-GA	0.93 (0.60–1.44)	0.737
	Recessive	GA-AA	1.00	
		GG	0.74 (0.30–1.81)	0.506
rs3203295	Genotype	AA	1.00	
		CC	0.70 (0.38–1.29)	0.253
		CA	0.75 (0.46–1.23)	0.255
	Additive	-	0.83 (0.61–1.11)	0.211
	Dominant	AA	1.00	
		CC-CA	0.73 (0.47–1.16)	0.187
	Recessive	CA-AA	1.00	
		CC	0.82 (0.48-1.41)	0.478
rs7928675	Genotype	AA	1.00	
		CC	1.63 (0.86–3.08)	0.135
		CA	1.04 (0.64–1.69)	0.874
	Additive	-	1.23 (0.91–1.67)	0.185
	Dominant	AA	1.00	
		CC-CA	1.18 (0.75–1.86)	0.462
	Recessive	CA-AA	1.00	
		CC	1.59 (0.89–2.86)	0.119
rs10832681	Genotype	GG	1.00	
		AA	1.07 (0.51–2.25)	0.862
		AG	1.74 (1.09–2.79)	0.020*
	Additive	-	1.23 (0.88–1.73)	0.230
	Dominant	GG	1.00	
		AA-AG	1.60 (1.02–2.50)	0.041*
	Recessive	AG-GG	1.00	
		AA	0.78 (0.39–1.56)	0.485

Notes: *$p < 0.05$ indicates statistical significance.

Abbreviations: SNP, single nucleotide polymorphism; OR, odds ratio; CI, confidence interval.

Table S8: Association between four candidate SNPs of *SOX6* and KBD risk (Number of affected joints stratified analysis)

SNP ID	Models	Genotype	Number of affected joints > 5 vs. Number of affected joints ≤ 5	
			OR (95% CI)	*p*
rs4539287	Genotype	AA	1.00	
		GG	0.83 (0.32–2.15)	0.70
		GA	0.74 (0.47–1.17)	0.199
	Additive	-	0.82 (0.57–1.18)	0.284
	Dominant	AA	1.00	
		GG-GA	0.75 (0.48–1.17)	0.206
	Recessive	GA-AA	1.00	
		GG	0.98 (0.39–2.44)	0.957
rs3203295	Genotype	AA	1.00	
		CC	0.97 (0.53–1.78)	0.93
		CA	1.40 (0.85–2.31)	0.186
	Additive	-	1.03 (0.76–1.40)	0.834
	Dominant	AA	1.00	
		CC-CA	1.25 (0.79–1.97)	0.347
	Recessive	CA-AA	1.00	
		CC	0.81 (0.47–1.40)	0.457
rs7928675	Genotype	AA	1.00	
		CC	0.96 (0.52–1.78)	0.894
		CA	1.29 (0.79–2.12)	0.307
	Additive	-	1.03 (0.76–1.39)	0.875
	Dominant	AA	1.00	
		CC-CA	1.18 (0.75–1.86)	0.474
	Recessive	CA-AA	1.00	
		CC	0.84 (0.48–1.48)	0.544
rs10832681	Genotype	GG	1.00	
		AA	0.73 (0.35–1.52)	0.399
		AG	1.32 (0.83–2.12)	0.244
	Additive	-	0.98 (0.70–1.38)	0.925
	Dominant	GG	1.00	
		AA-AG	1.19 (0.76–1.86)	0.460
	Recessive	AG-GG	1.00	
		AA	0.62 (0.31–1.24)	0.178

Notes: *$p < 0.05$ indicates statistical significance.

Abbreviations: SNP, single nucleotide polymorphism; OR, odds ratio; CI, confidence interval.

Table S9: Association between four candidate SNPs of *SOX6* and KBD risk (Grade-stratified analysis)

SNP ID	Models	Genotype	Grade					
			OR (95% CI)	p	OR (95% CI)	p	OR (95% CI)	p
			II vs I		III vs I		III vs II	
rs4539287	Genotype	AA	1.00		1.00		1.00	
		GG	0.73 (0.20–2.67)	0.633	1.02 (0.28–3.68)	0.979	1.34 (0.49–3.63)	0.570
		GA	1.04 (0.57–1.88)	0.898	0.55 (0.29–1.05)	0.069	0.51 (0.31–0.84)	0.008*
	Additive	—	0.96 (0.58–1.57)	0.859	0.76 (0.46–1.24)	0.265	0.76 (0.51–1.14)	0.181
	Dominant	AA	1.00		1.00		1.00	
		GG-GA	1.01 (0.56–1.80)	0.979	0.60 (0.32–1.11)	0.104	0.57 (0.35–0.93)	0.024*
	Recessive	GA-AA	1.00		1.00		1.00	
		GG	0.71 (0.20–2.49)	0.596	1.36 (0.39–4.71)	0.632	1.87 (0.71–4.92)	0.205
rs3203295	Genotype	AA	1.00		1.00		1.00	
		CC	1.27 (0.57–2.82)	0.565	1.24 (0.53–2.93)	0.622	0.98 (0.50–1.90)	0.947
		CA	1.13 (0.59–2.15)	0.715	1.10 (0.56–2.18)	0.783	0.98 (0.57–1.68)	0.934
	Additive	—	1.13 (0.76–1.67)	0.556	1.11 (0.73–1.69)	0.619	0.99 (0.71–1.37)	0.939
	Dominant	AA	1.00		1.00		1.00	
		CC-CA	1.17 (0.64–2.13)	0.607	1.14 (0.61–2.16)	0.680	0.98 (0.59–1.61)	0.929
	Recessive	CA-AA	1.00		1.00		1.00	
		CC	1.18 (0.58–2.43)	0.645	1.18 (0.54–2.55)	0.679	0.99 (0.54–1.80)	0.973
rs7928675	Genotype	AA	1.00		1.00		1.00	
		CC	0.62 (0.27–1.43)	0.264	0.99 (0.42–2.35)	0.976	1.43 (0.72–2.82)	0.308
		CA	0.69 (0.36–1.32)	0.260	0.87 (0.44–1.73)	0.693	1.24 (0.72–2.12)	0.437
	Additive	—	0.77 (0.52–1.16)	0.209	0.97 (0.64–1.48)	0.906	1.20 (0.86–1.67)	0.280
	Dominant	AA	1.00		1.00		1.00	
		CC-CA	0.67 (0.37–1.23)	0.195	0.90 (0.48–1.72)	0.759	1.29 (0.78–2.13)	0.314
	Recessive	CA-AA	1.00		1.00		1.00	
		CC	0.76 (0.36–1.61)	0.478	1.06 (0.49–2.32)	0.877	1.28 (0.69–2.37)	0.443
rs10832681	Genotype	GG	1.00		1.00		1.00	
		AA	0.64 (0.25–1.63)	0.346	0.81 (0.29–2.24)	0.686	1.23 (0.53–2.88)	0.630
		AG	0.75 (0.41–1.38)	0.356	1.20 (0.62–2.32)	0.589	1.57 (0.93–2.62)	0.089
	Additive	—	0.78 (0.51–1.20)	0.263	0.98 (0.61–1.58)	0.941	1.25 (0.86–1.82)	0.244
	Dominant	GG	1.00		1.00		1.00	
		AA-AG	0.73 (0.40–1.30)	0.283	1.12 (0.59–2.11)	0.731	1.50 (0.91–2.48)	0.109
	Recessive	AG-GG	1.00		1.00		1.00	
		AA	0.74 (0.31–1.78)	0.506	0.73 (0.28–1.86)	0.505	0.95 (0.43–2.11)	0.904

Notes: *$p < 0.05$ indicates statistical significance.

Abbreviations: SNP, single nucleotide polymorphism; OR, odds ratio; CI, confidence interval.

Table S10: Association between four candidate SNPs of *SOX6* and KBD risk (Hypertensive complications stratified analysis)

SNP ID	Models	Genotype	Hypertension vs Non-hypertensive (130 vs 222)	
			**OR (95% CI)**	***p***
rs4539287	Genotype	AA	1.00	
		GG	1.74 (0.67–4.51)	0.252
		GA	1.07 (0.66–1.71)	0.791
	Additive	-	1.19 (0.81–1.73)	0.380
	Dominant	AA	1.00	
		GG-GA	1.13 (0.71–1.79)	0.608
	Recessive	GA-AA	1.00	
		GG	1.68 (0.67–4.21)	0.264
rs3203295	Genotype	AA	1.00	
		CC	0.63 (0.33–1.20)	0.158
		CA	0.78 (0.47–1.29)	0.331
	Additive	-	0.79 (0.58–1.08)	0.141
	Dominant	AA	1.00	
		CC-CA	0.73 (0.45–1.16)	0.183
	Recessive	CA-AA	1.00	
		CC	0.72 (0.40–1.29)	0.269
rs7928675	Genotype	AA	1.00	
		CC	1.91 (0.99–3.65)	0.052
		CA	1.46 (0.87–2.46)	0.155
	Additive	-	1.39 (1.01–1.91)	0.043*
	Dominant	AA	1.00	
		CC-CA	1.58 (0.97–2.57)	0.064
	Recessive	CA-AA	1.00	
		CC	1.55 (0.86–2.77)	0.142
rs10832681	Genotype	GG	1.00	
		AA	1.50 (0.69–3.25)	0.305
		AG	0.96 (0.59–1.58)	0.878
	Additive	-	1.13 (0.79–1.62)	0.489
	Dominant	GG	1.00	
		AA-AG	1.04 (0.65–1.67)	0.866
	Recessive	AG-GG	1.00	
		AA	1.53 (0.75–3.15)	0.245

Note: *p < 0.05 indicates statistical significance.

Abbreviations: SNP, single nucleotide polymorphism; OR, odds ratio; CI, confidence interval.

Table S11: Association between four candidate SNPs of *SOX6* and KBD risk (Diabetes complications stratified analysis)

SNP ID	Models	Genotype	Diabetes vs Non- diabetes (20 vs 332)	
			**OR (95% CI)**	***p***
rs4539287	Genotype	AA	1.00	
		GG	1.02 (0.20–5.27)	0.980
		GA	0.43 (0.15–1.19)	0.105
	Additive	-	0.67 (0.30–1.48)	0.323
	Dominant	AA	1.00	
		GG-GA	0.50 (0.19-1.28)	0.147
	Recessive	GA-AA	1.00	
		GG	1.48 (0.30–7.31)	0.631
rs3203295	Genotype	AA	1.00	
		CC	0.68 (0.13–3.53)	0.644
		CA	1.90 (0.67–5.37)	0.224
	Additive	-	1.00 (0.53–1.88)	0.994
	Dominant	AA	1.00	
		CC-CA	1.51 (0.55–4.13)	0.423
	Recessive	CA-AA	1.00	
		CC	0.46 (0.10–2.09)	0.317
rs7928675	Genotype	AA	1.00	
		CC	0.44 (0.09–2.25)	0.327
		CA	1.03 (0.38–2.77)	0.955
	Additive	-	0.76 (0.39–1.48)	0.417
	Dominant	AA	1.00	
		CC-CA	0.85 (0.33–2.19)	0.731
	Recessive	CA-AA	1.00	
		CC	0.44 (0.10–2.01)	0.289
rs10832681	Genotype	GG	1.00	
		AA	1.11 (0.27–4.51)	0.888
		AG	0.61 (0.22–1.67)	0.338
	Additive	-	0.89 (0.43–1.85)	0.764
	Dominant	GG	1.00	
		AA-AG	0.70 (0.27–1.77)	0.447
	Recessive	AG-GG	1.00	
		AA	1.44 (0.39–5.36)	0.583

Note: *p < 0.05 indicates statistical significance.

Abbreviations: SNP, single nucleotide polymorphism; OR, odds ratio; CI, confidence interval.

Table S12: FPRP analysis of positive results of the overall analysis and subgroup analyses

SNP ID	Models	Genotype	OR (95%CI)	*p*	Statistical Power [a] (%)	Prior probability level		
						0.250	0.100	0.010
**Overall analysis**								
rs7928675	Allele	C/A	0.78 (0.64–0.97)	0.022	0.921	0.077	0.199	0.733
	Genotype	CC/AA	0.66 (0.43–1.00)	0.049	0.481	0.238	0.483	0.911
		CA/AA	0.63 (0.45–0.87)	0.006	0.366	0.040	0.110	0.576
	Additive	-	0.78 (0.64–0.96)	0.020	0.931	0.058	0.155	0.669
	Dominant	CC-CA/AA	0.64 (0.47–0.87)	0.004	0.397	0.032	0.090	0.522
rs10832681	Allele	A/G	0.79 (0.64–0.97)	0.028	0.947	0.072	0.188	0.718
	Genotype	AA/GG	0.57 (0.35–0.93)	0.023	0.265	0.216	0.453	0.901
	Additive	-	0.75 (0.60–0.94)	0.013	0.847	0.042	0.117	0.594
	Dominant	AA-AG/GG	0.71 (0.52–0.96)	0.027	0.659	0.106	0.263	0.797
Subgroup analysis (age ≤ 65)								
rs7928675	Genotype	CA/AA	0.57 (0.37–0.89)	0.013	0.245	0.141	0.330	0.844
	Dominant	CC-CA/AA	0.60 (0.40–0.91)	0.015	0.310	0.136	0.320	0.838
rs10832681	Genotype	AG/GG	0.62 (0.40–0.95)	0.029	0.451	0.734	0.892	0.989
	Additive	–	0.72 (0.53–0.99)	0.040	0.682	0.160	0.363	0.862
	Dominant	AA-AG/GG	0.62 (0.41–0.93)	0.021	0.363	0.147	0.341	0.850
Subgroup Analysis (Male)								
rs3203295	Genotype	CC/AA	1.96 (1.07–3.57)	0.029	0.191	0.304	0.567	0.935
	Additive	–	1.35 (1.01–1.80)	0.041	0.764	0.138	0.325	0.841
	Recessive	CC/CA-AA	1.80 (1.04–3.12)	0.037	0.258	0.296	0.558	0.933
rs7928675	Genotype	CA/AA	0.55 (0.34–0.88)	0.012	0.211	0.152	0.350	0.856
	Additive	–	0.73 (0.55–0.97)	0.030	0.734	0.109	0.269	0.802
	Dominant	CC-CA/AA	0.56 (0.36–0.86)	0.008	0.213	0.102	0.254	0.790
rs10832681	Genotype	AA/GG	0.47 (0.23–0.97)	0.042	0.172	0.417	0.682	0.959
	Additive	–	0.71 (0.52–0.98)	0.039	0.649	0.147	0.341	0.850
subgroup analysis (BMI ≥ 24)								
rs10832681	Genotype	AG/GG	0.50 (0.29–0.85)	0.010	0.144	0.179	0.395	0.878
	Dominant	AA-AG/GG	0.55 (0.33–0.91)	0.020	0.227	0.209	0.442	0.897
Subgroup analysis (BMI < 24)								
rs3203295	Genotype	CC/AA	2.16 (1.24–3.74)	0.006	0.096	0.157	0.358	0.860
	Additive	–	1.42 (1.09–1.86)	0.009	0.655	0.048	0.130	0.622
	Recessive	CC/CA-AA	1.96 (1.19–3.23)	0.008	0.147	0.145	0.336	0.848
rs7928675	Genotype	CA/AA	0.53 (0.34–0.83)	0.005	0.158	0.095	0.240	0.776
	Dominant	CC-CA/AA	0.57 (0.38–0.86)	0.008	0.228	0.089	0.226	0.763
rs10832681	Genotype	AA/GG	0.49 (0.25–0.94)	0.032	0.177	0.350	0.618	0.947
	Recessive	AA/AG-GG	0.53 (0.29–0.96)	0.038	0.225	0.326	0.592	0.941
Subgroup analysis (Smoking, yes)								
rs10832681	Genotype	AA/GG	0.40 (0.17–0.96)	0.041	0.126	0.488	0.741	0.969
	Additive	–	0.66 (0.45–0.98)	0.041	0.480	0.197	0.425	0.890

(Continued)

Table S12: *Continued*

SNP ID	Models	Genotype	OR (95%CI)	*p*	Statistical Power [a] (%)	Prior probability level		
						0.250	0.100	0.010
**Overall analysis**								
Subgroup analysis (Smoking, no)								
rs7928675	Genotype	CC/AA	0.57 (0.34–0.96)	0.035	0.278	0.272	0.528	0.925
		CA/AA	0.59 (0.39–0.90)	0.014	0.285	0.131	0.311	0.832
	Additive	–	0.73 (0.57–0.95)	0.019	0.750	0.071	0.187	0.717
	Dominant	CC-CA/AA	0.59 (0.40–0.87)	0.008	0.269	0.080	0.206	0.741
Subgroup analysis (Course of disease > 38 vs. Course of disease ≤ 38)								
rs10832681	Genotype	AG/GG	1.74 (1.09–2.79)	0.020	0.269	0.193	0.418	0.888
	Dominant	AA-AG/GG	1.60 (1.02–2.50)	0.041	0.388	0.232	0.475	0.909
Subgroup analysis (Grade Ⅲ vs. Ⅱ)								
rs4539287	Genotype	GA/AA	0.51 (0.31–0.84)	0.008	0.146	0.143	0.334	0.847
	Dominant	GG-GA/AA	0.57 (0.35–0.93)	0.024	0.265	0.216	0.453	0.901
Subgroup analysis (Hypertension vs Non-hypertensive)								
rs7928675	Additive	–	1.39 (1.01–1.91)	0.043	0.681	0.157	0.358	0.860

Notes: *p < 0.05 indicates statistical significance.

Abbreviations: SNP, single nucleotide polymorphism; OR, odds ratio; CI, confidence interval; FPRP, false-positive report probability.
